# Clinical efficacy and prognosis analysis of treatment regimens for *EGFR* mutant non-small cell lung cancer and brain metastasis: a retrospective study

**DOI:** 10.1186/s12885-023-10744-2

**Published:** 2023-03-30

**Authors:** Huijuan Wang, Ruyue Xing, Mengmeng Li, Mina Zhang, Chunhua Wei, Guowei Zhang, Yuanyuan Niu, Zhiyong Ma, Xiangtao Yan

**Affiliations:** Department of Medical Oncology, The Affiliated Cancer Hospital of Zhengzhou University, Henan Cancer Hospital, No. 127 Dongming Road, Zhengzhou, 450000 China

**Keywords:** NSCLC, Tyrosine kinase inhibitors, Bevacizumab, Brain metastasis, Intracranial metastasis

## Abstract

**Background:**

The aims of the study were to evaluate potential differences among first-line treatment for *EGFR* mutant (m+) non-small cell lung cancer (NSCLC) patients with brain metastasis in China and to identify the factors influencing survival outcomes.

**Methods:**

In this retrospective study, 172 *EGFR*m + patients with advanced NSCLC who received a 1st generation EGFR tyrosine kinase inhibitor (TKI) were divided into 4 groups: A, EGFR-TKI (n = 84); B, EGFR-TKI + pemetrexed + cisplatin/carboplatin chemotherapy (CT) (n = 55); C, EGFR-TKI + bevacizumab (n = 15); and D, EGFR-TKI + pemetrexed + cisplatin/carboplatin CT + bevacizumab (n = 18). Intracranial and extracranial progression-free survival (PFS), the overall survival (OS), objective remission rates (ORRs) and adverse events were analyzed.

**Results:**

Intracranial PFS of groups C + D was longer than for groups A + B (18.9 m vs. 11.0 m, *P* = 0.027). Extracranial PFS were longer in group B in comparison with group A (13.0 m vs. 11.5 m, *P* = 0.039) and in groups C + D compared to groups A + B (18.9 m vs. 11.9 m, *P* = 0.008). Median OS in groups A and B were 27.9 m and 24.4 m, respectively, while groups C and D have not yet achieved median OS. Significant difference was found in intracranial ORR between groups A + B vs. C + D (31.0% vs. 65.2%, *P* = 0.002). Most patients suffered grade 1–2 treatment-related adverse events, which were relieved soon after symptomatic treatment.

**Conclusions:**

First-generation EGFR-TKI + bevacizumab treatment outperformed other regimens in *EGFR*m + NSCLC patients with brain metastasis. The therapy improved the control and delayed progression of intracranial lesions and prolonged survival times.

**Supplementary Information:**

The online version contains supplementary material available at 10.1186/s12885-023-10744-2.

## Background

Non-small cell lung cancer (NSCLC) patients have unacceptable morbidity and mortality rates [1], with only 15% surviving for up to 5 years after diagnosis [2]. Epidermal growth factor receptor (*EGFR*) mutations, are known oncogenic drivers in NSCLC patients and east Asian individuals with NSCLC had a substantially greater *EGFR* mutant (m +) prevalence than Caucasian patients (about 30% vs. 7%, respectively) [3]. In China the prevalence of *EGFR*m + NSCLC cases has been estimated to be 36.5–40.3% [4]. According to the PIONEER study, from 372 Chinese NSLC *EGFR*m + patients, 346 had *EGFR* activating mutations, with 182 exon 19 deletions and 169 L858R point mutations being the most common mutation types [5]. About 70% of *EGFR*m+ NSCLC patients develop brain metastases (BMs), compared to an incidence of 38% of NSCLC cases without an *EGFR* wild-type mutation [6]. The percentage of patients diagnosed with advanced NSCLC and BMs is about 25–30% on first diagnosis and a further 40–50% develop BMs during the subsequent course of the disease [7]. Although third-generation tyrosine kinase inhibitors (TKIs) that target EGFR have enhanced central nervous system (CNS) permeability and show better CNS efficacy in patients compared to first-generation EGFR-TKIs, the limited treatment options after drug resistance highlights the urgent need for alternative treatment strategies for patients with BMs [8, 9]. It is noteworthy that most NSCLC patients with BMs are not usually included in clinical trials. Therefore, the best first-line therapy for *EGFR*m + NSCLC with BMs has yet to be unequivocally established.

In this real-world clinical study, systemic and local treatment outcomes of patients with BMs were retrospectively analyzed focusing on *EGFR*m + NSCLC combined with BMs cases and included 1st generation EGFR-TKI treatment alone and 1st generation EGFR-TKI plus chemotherapy or anti-angiogenesis drugs. The patients may or may not have received brain radiotherapy. This allowed us to evaluate potential differences among first-line treatments for *EGFR*m + NSCLC patients with BMs and provide a useful reference source for effective future clinical applications.

## Methods

### Patients

**Fig. 1:**
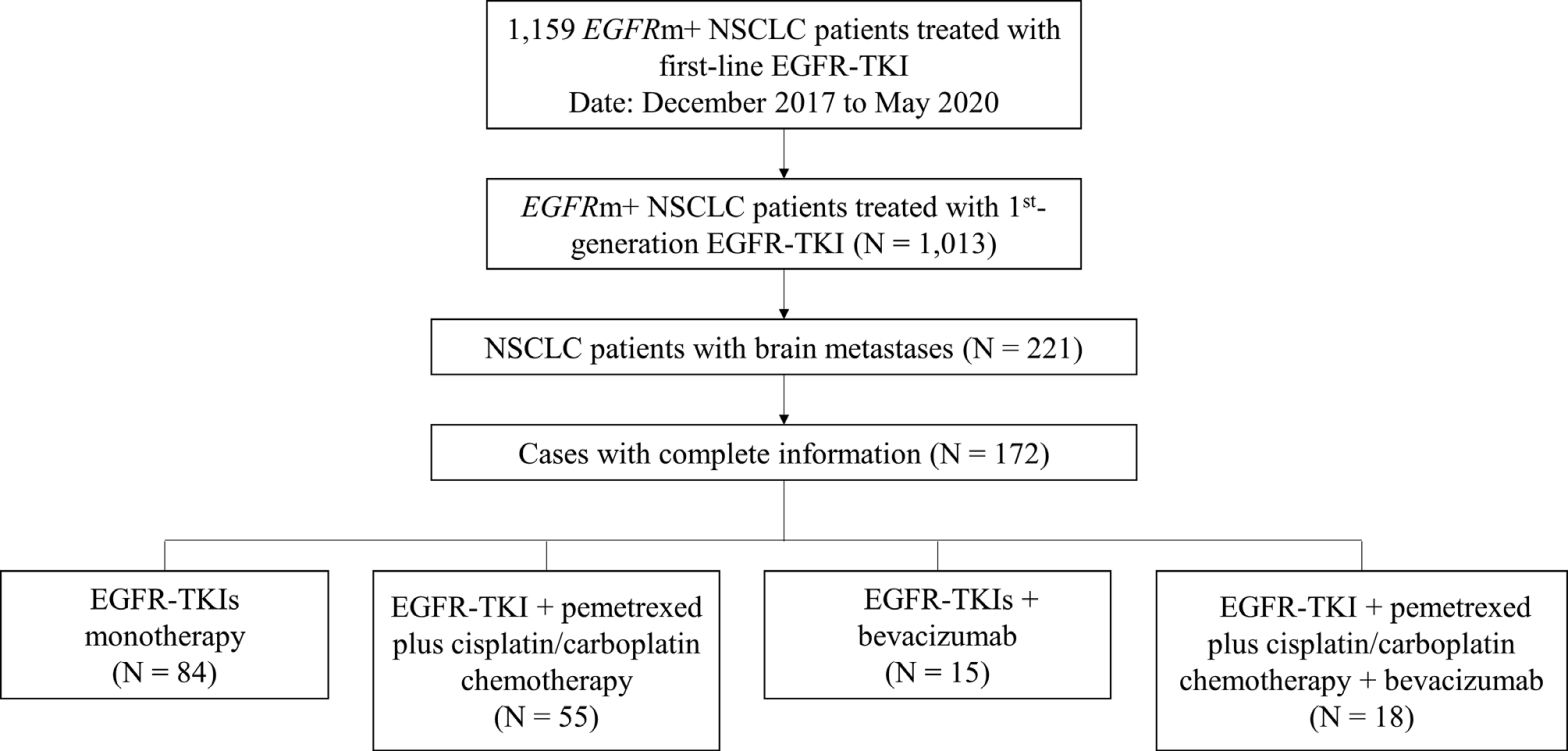
Flowchart of the study

In this retrospective study, 1,159 Chinese patients with advanced *EGFR*m + NSCLC who received first-line EGFR-TKI treatment at Zhengzhou University Cancer Hospital from December 2017 to May 2020 were screened, including 1,013 patients who received 1st generation EGFR-TKI treatment, of whom 221 patients (21.8%) with newly diagnosed BMs, of whom 172 finished their treatment regimen and had complete follow-up data, were selected for the analysis (Fig. 1). The inclusion criteria were: (1) NSCLC confirmed cytologically or histologically; (2) *EGFR* mutations verified by Amplification Refractory Mutation System PCR or next generation sequencing by analyzing histological and cytological specimens obtained from primary and metastatic lesions; (3) First-line treatment after diagnosis was 1st generation EGFR-TKIs or 1st generation EGFR-TKIs combination therapy; (4) Patients had brain MRI scans before being given EGFR-TKIs therapy; and (5) Complete treatment process and follow-up data were available.

The study was approved by the Ethics Committee of the Affiliated Cancer Hospital of Zhengzhou University, Henan Cancer Hospital and was conducted strictly following the Declaration of Helsinki of the World Medical Association guidelines. Patient consent was waived due to the nature of the retrospective study.

### Treatment regimens

Clinical data from 172 *EGFR*m + NSCLC patients with BMs were retrospectively analyzed and according to different first-line treatment regimens, they were allocated to 1 of 4 groups: A, 84 patients who received 1st generation EGFR-TKI monotherapy; B, 55 patients given 1st generation EGFR-TKIs + platinum-containing chemotherapy; C, 15 treated with 1st generation EGFR-TKIs combined with anti-angiogenic drugs; and D, 18 treated with 1st generation EGFR-TKIs + platinum-containing chemotherapy + anti-angiogenic drugs.

The first-generation EGFR-TKIs included gefitinib (250 mg once daily (qd)) and icotinib (125 mg 3 times a day (tid)). In combination therapy, chemotherapy regimens were pemetrexed (500 mg/m^2^ d1) combined with cisplatin (75 mg/m^2^ d1)/carboplatin (AUC 5 d1). The anti-angiogenic drug was bevacizumab (7.5 mg/kg d1), 21 days per cycle, a total of 4 cycles, then pemetrexed plus bevacizumab maintenance.

Patients were given EGFR-TKIs or EGFR-TKI combination treatment until the disease progressed or unacceptable toxicity occurred. The primary endpoints were the median overall progression-free survival (PFS), median intracranial PFS (iPFS) and median extracranial PFS (ePFS); and the secondary endpoints overall survival (OS), intracranial objective remission rates (ORRs), extracranial ORRs and adverse events.

### Data collection and clinical efficacy evaluation

This study retrospectively collected patients’ data based on different first-line treatment regimens and data from intrapulmonary tumors, intracranial metastases, and other metastases obtained from enhanced CT and/or MRI images evaluated. To determine the factors influencing survival of *EGFR*m + NSCLC patients with BMs, intracranial and extracranial objective response rates, iPFS and median OS values were compared among all groups.

PFS was the period of time from EGFR-TKI therapy until progressive disease (PD) or death. OS was the period from the onset of EGFR-TKI treatment or death. ePFS was the period from any EGFR-TKI therapy to the onset of extracranial PD (other than intracranial PD) or death. iPFS was defined as the period from EGFR-TKI treatment to intracranial PD or death. Response assessment criteria were used for solid tumors thus: partial response (PR); stable disease (SD); complete response (CR); and PD, based on RECIST ver. 1.1 [10]. Patients in each group were followed-up by regular reexaminations (blood routine and tumor marker tests, ultrasound examination, MRI examination, nuclear medicine examination, tissue or cytology examination) 1 month after the first EGFR-TKI treatment and 2 months after each subsequent treatment, and also by telephone follow-ups.

### Statistical analysis

SPSS ver. 26.0 was employed to analyze data. A chi-squared or Fisher’s exact test was used for feature comparison. The Kaplan-Meier method was employed to determine PFS (months) and OS (months). Survival data were plotted using GraphPad Prism ver. 7.03. Independent factors were evaluated using a Cox regression model. *P* < 0.05 was considered to be significant at the bilateral level.

## Results

### Clinical characteristics of the enrolled patients

Table 1 shows the baseline characteristics of patients. Overall, the median age was 60 years (range: 31–82). The majority were female (61.0%), had no smoking history (78.5%), had an ECOG score of 0–1 (77.9%), and had no neurological symptoms (66.9%). Most *EGFR* mutations in patients were exon 19 deficiency mutations (52.9%) and only 39 patients (22.7%) received brain radiotherapy. Among these 39 patients, 20 received stereotype radiosurgery (SRS), and 19 received whole brain cranial irradiation (WBRT) who had previously cranial radiation, 5 received upfront cranial irradiation, 24 received radiations concurrent with first-line targeted therapy and 10 received radiation after brain progression. The 5 patients who received upfront cranial irradiation were excluded from the efficacy analysis since upfront cranial irradiation might lead to better survival outcomes in patients with *EGFR*m + NSCLC with BMs [11]. No differences were detected in the baseline characteristics between groups A and B, C and D, or groups not treated with bevacizumab (A + B) or treated with bevacizumab (C + D) (**Supplementary Table 1**).

**Table 1:** Baseline characteristics of patients

Characteristic	N	Group A	Group B	*P*1	Group C	Group D	*P*2
	(n = 172)	(n = 84)	(n = 55)		(n = 15)	(n = 18)	
**Age (years)**				0.393			0.418
Median (range)	60 (31–82)	62 (32–82)	57 (31–78)		67 (36–79)	58 (46–72)	
< 70	142 (82.6%)	67 (79.8%)	47 (85.5%)		10 (66.7%)	15 (83.3%)	
≥ 70	30 (17.4%)	17 (20.2%)	8 (14.5%)		5 (33.3%)	3 (16.7%)	
**Gender**				0.315			0.488
Male	67 (39.0%)	31 (36.9%)	25 (45.5%)		6 (40.0%)	5 (27.8%)	
Female	105 (61.0%)	53 (63.1%)	30 (54.5%)		9 (60.0%)	13 (72.2%)	
**Smoking status**				0.230			1.000
Yes	37 (21.5%)	17 (20.2%)	16 (29.1%)		2 (13.3%)	2 (11.1%)	
No	135 (78.5%)	67 (79.8%)	39 (70.9%)		13 (86.7%)	16 (88.9%)	
**ECOG PS**				0.825			0.346
0–1	134 (77.9%)	64 (76.2%)	41 (74.5%)		14 (93.3%)	14 (77.8%)	
2	38 (22.1%)	20 (23.8%)	14 (25.5%)		1 (6.7%)	4 (22.2%)	
***EGFR *** **mutation type**				0.365			0.739
Deletion in exon 19	91 (52.9%)	46 (54.8%)	24 (43.6%)		9 (60.0%)	12 (66.7%)	
21 L858R	72 (41.9%)	34 (40.4%)	29 (52.7%)		5 (33.3%)	4 (22.2%)	
Others	9 (5.2%)	4 (4.8%)	2 (3.7%)		1 (6.7%)	2 (11.1%)	
**Neurologic symptoms**				0.334			1.000
Yes	57 (33.1%)	24 (28.6%)	20 (36.4%)		6 (40.0%)	7 (38.9%)	
No	115 (66.9%)	60 (71.4%)	35 (63.6%)		9 (60.0%)	11 (61.1%)	
**Cranial radiation**				0.393			0.773
SRS	20 (11.6%)	8 (9.5%)	5 (9.1%)		4 (26.7%)	3 (16.7%)	
WBRT	19 (11.0%)	9 (10.7%)	3 (5.5%)		3 (20.0%)	4 (22.2%)	
No	133 (76.3%)	67 (79.8%)	47 (85.5%)		8 (53.3%)	11 (61.1%)	
**1st generation EGFR-TKIs**				0.323			1.000
Gefitinib	123 (71.5%)	56 (66.7%)	41 (74.5%)		12 (80.0%)	14 (77.8%)	
Icotinib	49 (28.5%)	28 (33.3%)	14 (25.5%)		3 (20.0%)	4 (22.2%)	

Note: Group A: 1st generation EGFR-TKIs monotherapy; Group B: 1st generation EGFR-TKIs + pemetrexed plus cisplatin/carboplatin chemotherapy; Group C: 1st generation EGFR-TKIs + bevacizumab; Group D: 1st generation EGFR-TKIs + pemetrexed plus cisplatin/carboplatin chemotherapy + bevacizumab

Abbreviations: SRS, stereotactic radiosurgery; TKI, tyrosine kinase inhibitor; WBRT, whole-brain radiation therapy

### Efficacy analysis

The therapeutic effects of both intracranial and extracranial lesions were evaluated in 172 patients, of which intracranial therapeutic effects were evaluated in 139 patients and extracranial therapeutic effects in 128 patients.

#### Primary endpoints

##### Overall PFS times

**Fig. 2:**
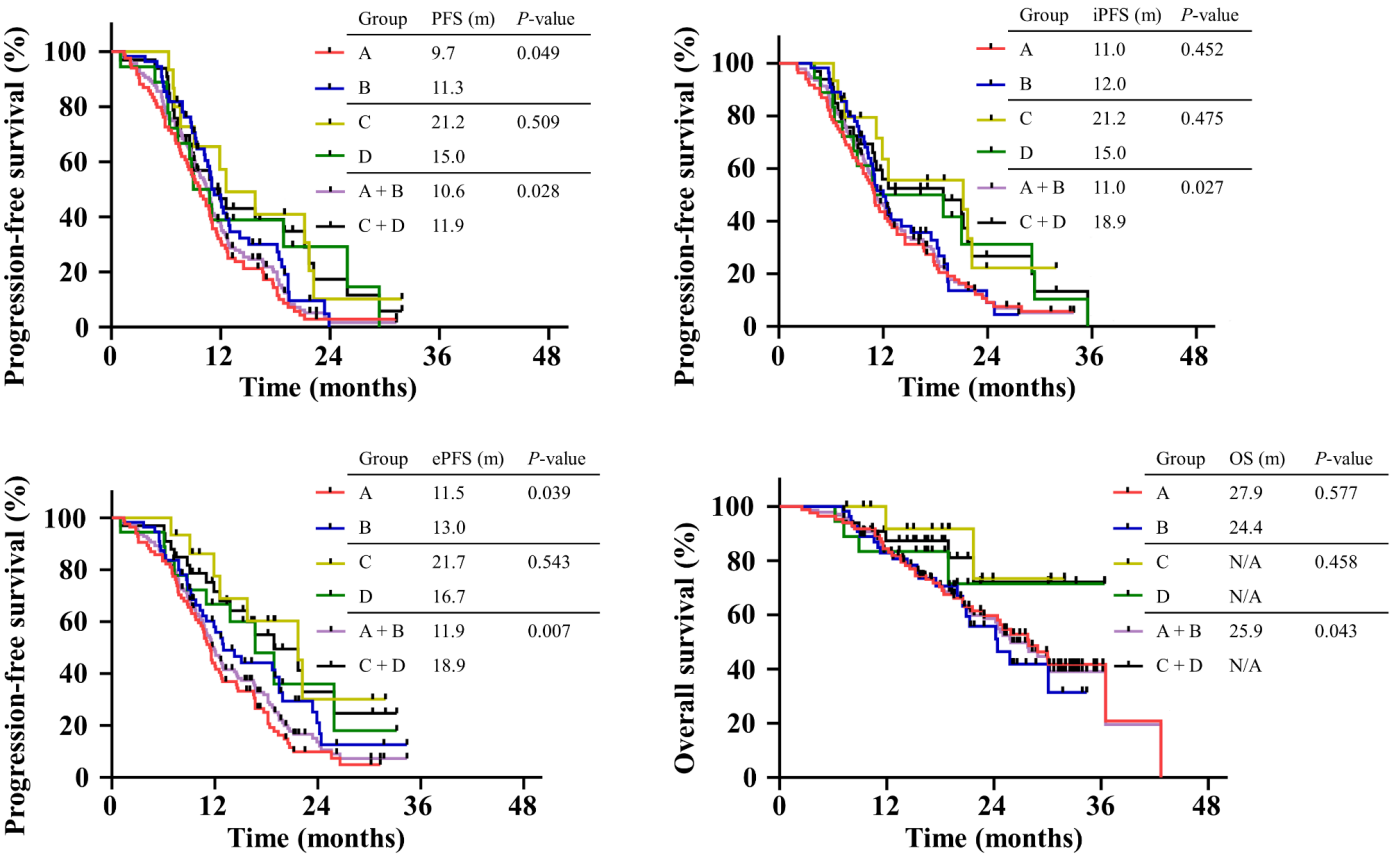
**PFS, iPFS, ePFS and OS comparison in*EGFR*m + NSCLC patients with BMs under different regimens.** BMs, brain metastases; *EGFR*m+, *EGFR*-mutant positive; m, month; PFS: progression-free survival; OS, overall survival; iPFS, intracranial progression-free survival; ePFS, extracranial progression-free survival

Group B showed longer overall PFS than group A (9.7 vs. 11.3, *P =* 0.049). The comparison of overall PFS revealed no differences between group C and group D (12.5 vs. 9.9, *P* = 0.509). In comparison to group A + B, the overall PFS time in the group C + D was significantly longer (11.9 vs. 10.6, *P* = 0.028) (Table 2; Fig. 2).

**Table 2:** Comparison of primary endpoints among different regimes

Therapeutic regimen	Median PFS	95% CI	*P* value	Median iPFS	95% CI	*P* value	Median ePFS	95% CI	*P* value
Group A	9.7 m	8.2–11.1	0.049	11.0 m	9.7–12.3	0.452	11.5 m	10.5–12.3	0.039
Group B	11.3 m	9.8–12.7		12.0 m	10.4–13.5		13.0 m	9.5–16.5	
Group C	12.5 m	6.1–19.0	0.509	21.2 m	8.9–33.6	0.475	21.7 m	14.7–28.7	0.543
Group D	9.9 m	4.7–13.3		15.0 m	7.8–29.9		16.7 m	10.7–22.5	
Group A + B	10.6 m	9.7–11.5	0.028	11.0 m	10.0-12.1	0.027	11.9 m	10.6–13.2	0.008
Group C + D	11.9 m	7.5–11.7		18.9 m	9.3–31.5		18.9 m	12.6–25.2	

Note: Group A: 1st generation EGFR-TKIs monotherapy; Group B: 1st generation EGFR-TKIs + pemetrexed plus cisplatin/carboplatin chemotherapy; Group C: 1st generation EGFR-TKIs + bevacizumab; Group D: 1st generation EGFR-TKIs + pemetrexed plus cisplatin/carboplatin chemotherapy + bevacizumab

Abbreviations: ePFS, extracranial PFS; iPFS, intracranial PFS; PFS, progression-free survival; TKI, tyrosine kinase inhibitor

##### iPFS times

There was no significant difference between groups A and B (11.0 vs. 12.0, *P* = 0.452), or groups C and D (21.2 vs. 15.0, *P* = 0.475) groups, indicating that chemotherapy did not improve iPFS. Compared with the groups without bevacizumab (A + B), the iPFS time in groups with bevacizumab (C + D) was significantly longer (11.0 vs. 18.9, *P* = 0.027) (Table 2; Fig. 2).

##### ePFS times

The ePFS times in groups A, B, C and D were 11.5, 13.0, 21.7 and 16.7 months, respectively (Table 2). Significant differences were found in extracranial efficacy for groups A and B (*P* = 0.039) as well as between groups A + B vs. C + D (*P* = 0.008), indicating that single drug EGFR-TKIs regimens had inferior efficacy for controlling extracranial lesions compared to combination therapies. Furthermore, bevacicumab combination regimes effectively enhanced ePFS times (Table 2; Fig. 2).

#### Secondary endpoints

For 172 *EGFR* mutation NSCLC patients, 23.9 months was the median follow-up time (95% CI: 20.4–27.5) and the median OS times of group A and B were 27.9 months (95% CI: 22.6–33.2) and 24.4 months (95% CI: 18.5–30.2). Similarly, a median OS time of 25.9 months (95% CI: 22.0–29.9) was recorded for groups A + B, whereas group C + D still did not reach the median OS time, but it was already greater than in groups A + B by the most recent follow-up date (*P* = 0.043) **(**Table 3; Fig. 2).

**Table 3:** Comparison of secondary endpoints among different regimens

Therapeutic regimen	OS (m)	95% CI		*P* value		Intracranial ORR		*P* value	Extracranial ORR	*P* value
Group A	27.9	22.6–33.2	0.577		28.6% (21/75)		0.339		48.6% (35/74)	0.139
Group B	24.4	18.5–30.2			36.6% (15/41)				62.2% (23/37)	
Group C	N/A	-	0.458		66.7% (6/9)		1.000		71.4% (5/7)	1.000
Group D	N/A	-			64.3% (9/14)				80.0% (8/10)	
Group A + B	25.9	22.0-29.9	0.043		31.0% (36/116)		0.002		52.3% (58/111)	0.108
Group C + D	N/A	-			65.2% (15/23)				76.5% (13/17)	

Note: Group A: 1st generation EGFR-TKIs monotherapy; Group B: 1st generation EGFR-TKIs + pemetrexed plus cisplatin/carboplatin chemotherapy; Group C: 1st generation EGFR-TKIs + bevacizumab; Group D: 1st generation EGFR-TKIs + pemetrexed plus cisplatin/carboplatin chemotherapy + bevacizumab

Abbreviations: m, month; N/A, not reached; ORR, overall remission rate; OS, overall survival; TKI, tyrosine kinase inhibitor

In terms of ORR, a significant difference was found in intracranial ORR between groups A + B vs. C + D (31.0% vs. 65.2%, *P* = 0.002). However, there was no significant difference in extracranial ORR between A and B, or the C and D groups, or between groups A + B vs. C + D **(**Table 3).

### Analysis of risk factors affecting iPFS

Univariate Cox regression analyses of iPFS-related prognostic factors in patients showed that the factors that significantly affected iPFS (*P* < 0.05) included: whether bevacizumab was administered; intracranial symptoms; and sensitive mutations. Patients with sensitive mutations, who received first-line combination therapy with bevacizumab and without intracranial symptoms had longer iPFS times. Factors that showed a correlation after analysis using univariate Cox regression (*P* < 0.05) were carried into multivariate Cox regression. The results showed that first-line combination with bevacizumab (*P* = 0.015), patients with sensitive mutations (*P* = 0.002) and absence of intracranial symptoms (*P* = 0.014) were still independent factors of slow intracranial progression (Table 4).

**Table 4:** Univariate and multivariate analysis of iPFS

Independent risk factor		Univariate analysis of iPFS			Multivariate analysis of iPFS		
		*P* value	HR value	95% CI	*P* value	HR value	95% CI
**First line regimens**	Group C + D: Group A + B	0.029	0.599	0.378–0.949	0.015	0.563	0.355–0.895
	Group B: Group A	0.490	0.874	0.595–1.282			
**Age**	< 70: ≥ 70	0.990	0.997	0.646–1.539			
**Gender**	Female: male	0.985	0.997	0.706–1.407			
**Intracranial symptoms**	No: Yes	0.026	0.676	0.479–0.955	0.014	0.647	0.458–0.915
**Smoking**	No: Yes	0.362	0.825	0.545–1.248			
***EGFR *** **mutation**	Sensitive mutation: others	0.003	0.346	0.173–0.690	0.002	0.333	0.167–0.665
	21L858R :19DEL	0.778	1.052	0.741–1.493			
**Brain radiation therapy**	Concurrent radiotherapy: others	0.148	1.432	0.880–2.430			

Note: Group A: 1st generation EGFR-TKIs monotherapy; Group B: 1st generation EGFR-TKIs + pemetrexed plus cisplatin/carboplatin chemotherapy; Group C: 1st generation EGFR-TKIs + bevacizumab; Group D: 1st generation EGFR-TKIs + pemetrexed plus cisplatin/carboplatin chemotherapy + bevacizumab

Abbreviations: iPFS, intracranial progression-free survival; TKI, tyrosine kinase inhibitor

### Univariate/multivariable analyses of risk factors affecting ePFS

Patient data were further evaluated using univariate and multivariate analyses (*vide supra*) to investigate potential variables linked to ePFS. As shown in Table 5, the factors that significantly affected ePFS (*P* < 0.05) were first-line TKI regimens combined with bevacizumab, TKI combination therapy, and sensitive mutations. The factors that showed a correlation in univariate Cox regression analysis (*P* < 0.05) were carried into the multivariate analysis, and the results revealed that only a first-line TKI combination with bevacizumab was an independent factor for prolongation of ePFS times.

**Table 5:** Univariate and multivariate analysis of ePFS

Independent risk factor		Univariate analysis of ePFS			Multivariate analysis of ePFS		
		*P* value	HR value	95% CI	*P* value	HR value	95% CI
**First line regimens**	Group C + D: Group A + B	0.009	0.507	0.304–0.846	0.010	0.508	0.305–0.848
	Group B: Group A	0.038	0.658	0.442–0.978			
**Age**	< 70: ≥ 70	0.345	0.810	0.522–1.255			
**Gender**	Female: male	0.484	0.881	0.618–1.256			
**Intracranial symptoms**	No: Yes	0.275	1.233	0.846–1.795			
**Smoking**	No: Yes	0.159	0.741	0.489–1.124			
***EGFR *** **mutation**	Sensitive mutation: others	0.049	0.529	0.245–1.143	0.110	0.534	0.247–1.153
	19DEL: 21L858R	0.188	1.272	0.889–1.821			

Note: Group A: 1st generation EGFR-TKIs monotherapy; Group B: 1st generation EGFR-TKIs + pemetrexed plus cisplatin/carboplatin chemotherapy; Group C: 1st generation EGFR-TKIs + bevacizumab; Group D: 1st generation EGFR-TKIs + pemetrexed plus cisplatin/carboplatin chemotherapy + bevacizumab

Abbreviations: ePFS, extracranial progression-free survival; TKI, tyrosine kinase inhibitor

### Univariate and multivariate analyses of risk factors affecting OS

Patients were further analyzed by univariate and multivariate regression to evaluate the prognostic factors associated with OS. Factors that significantly affected OS (*P* < 0.05) were age, smoking status and sensitive *EGFR* mutations. Factors that showed a correlation in univariate Cox regression analysis (*P* < 0.05) were carried into the multivariate analysis, and the results revealed that age < 70 years was still an independent prognostic factors for improving OS (**Supplementary Table 2**).

### Adverse events

In this study, most patients suffered grade 1–2 treatment related adverse events, which were relieved soon after symptomatic treatment. The incidence of gastrointestinal reactions was significantly higher in group B than in group A (49.1% vs. 19.0%, *P* = 0.001), and significantly higher in group D than in group C (55.6% vs. 20.0%, *P* = 0.033). The incidence of myelosuppression was significantly higher in group B than in group A (45.5% vs. 0.0%, *P* = 0.001), and significantly higher in group D than in group C (44.4% vs. 0.0%, *P* = 0.001) (**Supplementary Table 3**).

## Discussion

Patients with BMs have a poor prognosis, and the rate of BMs if patients have an *EGFR* mutation is about 3 times greater than those without a mutation [12]. Since 2014, EGFR-TKI therapy has become the gold standard for first-line therapy of *EGFR*m + NSCLC [13–15]. However, as most EGFR-TKIs cannot pass the blood-brain barrier [8], the EGFR-TKIs in patients with brain lesions and BMs has limited efficacy, despite a good control rate of primary lesions.

The findings of the present analysis revealed that first-generation EGFR-TKI combined with an anti-angiogenesis drug significantly improved the efficacy of control of brain lesions, delayed the progression of intracranial lesions, improved prognosis and prolonged the survival times of patients with *EGFR*m + NSCLC with BMs. Although EGFR-TKIs plus concomitant chemotherapy improved the control of extracranial lesions compared with targeted therapy alone, it had limited efficacy for intracranial lesions and did not significantly enhance long-term survival of patients. For patients who received synchronous intracranial radiotherapy, although the control rate of intracranial lesions was improved, prolonged iPFS was not converted into longer OS times (data not shown).

In this retrospective study, patients who received 1st generation EGFR-TKIs combined with chemotherapy showed longer overall PFS than the 1st generation EGFR-TKIs monotherapy group (11.3 vs. 9.7, *P* = 0.049). Furthermore, patients who received 1st generation EGFR-TKIs alone had intracranial ORRs of 28.6%, while the extracranial ORR was up to 48.6%. Patients who received 1st generation EGFR-TKIs combined with chemotherapy had intracranial ORRs of 36.6%, while the extracranial ORR was up to 62.2%. These results are in good agreement with a previous NEJ009 study in which the ORRs and PFS times of the combination therapy groups were superior to the solely treated gefitinib TKI group (20.9 months vs. 17.5 months) [16]. Another JMIT study also reported the clinical value of administering gefitinib combined with pemetrexed, with the median PFS time being 15.8 months [17]. Preclinical studies have shown that cytotoxic synergies in NSCLC cell lines were observed when pemetrexed was applied in combination with EGFR-TKIs. EGFR-TKIs mainly play cytotoxic roles through G1 phase arrest, while pemetrexed works through S phase arrest, suggesting that the two can play a role in different tumor cell populations, thereby improving overall clinical efficacy [18]. Another advantage of combination chemotherapy is that as first-line treatment progresses, subsequent chemotherapy regimen can still be beneficial providing more options for long-term efficacy. However, this study evaluated the intracranial and extracranial efficacy separately for patients with BMs, and found that combined chemotherapy was more likely to improve the extracranial efficacy.

In the present study, EGFR-TKI combined with bevacizumab was the most beneficial regimen for both PFS and OS outcomes, with an iPFS time reaching 21.2 months and an ePFS time of 21.7 months, while the OS time had not reached the midpoint but far exceeded other treatment regimens. Preclinical studies have shown that concomitant inhibition of pathways involving EGFR and VEGF/VEGFR can produce biological synergies against tumor activity [19]. Other studies have shown that simultaneous inhibition of these pathways can prevent resistance to EGFR-TKIs, or resistance acquired because of the presence of *EGFR* T790M mutations [20, 21]. This synergistic effect has been further confirmed in a number of clinical trials including JO25567, BELIEF and NEJ026 [22–25]. In the BRAIN study, bevacizumab was shown to be effective against BMs, with a total intracranial response rate of 61.2% [26]. However, the synergistic effect of EGFR-TKI and bevacizumab on BMs has only been reported in some retrospective single-arm studies [27–29]. Therefore, further studies of the therapeutic effectiveness of this combination treatment in patients with BMs is required in order to provide more empirical support for the next large-scale prospective study.

It should be noted that the combination of EGFR-TKI with bevacizumab and pemetrexed did not achieve the desired effect, with an iPFS of 15.0 months and ePFS of 16.7 months, which might due to the small cohort of patients studied and the relatively brief duration of treatment. Although previous PointBreak and ECOG 5508 studies [30, 31] suggested that pemetrexed plus bevacizumab combined maintenance therapy was beneficial for PFS compared to pemetrexed or bevacizumab alone, it has also been mentioned that long-term use of pemetrexed in combination with bevacizumab in NSCLC may increase the risk of treatment-related toxicity. The decision to use EGFR-TKIs combined with pemetrexed and bevacizumab should be made with caution in view of the majority of patients with an *EGFR*-negative mutation in the previous study population and the potential adverse reactions and treatment costs associated with this regimen.

To date, third-generation EGFR-TKIs with good CNS permeability and efficacy (e.g. osimertinib) have produced better CNS results compared to first-generation EGFR-TKIs in *EGFR*m + patients with BMs. They have become one standard solution for the first-line therapy of advanced or metastatic *EGFR*m + NSCLC patients. A phase 1/2 single-group open-label trial showed that the combination therapy of osimertinib and bevacizumab achieved the preset primary efficacy endpoint without producing significant toxicity [32]. However, phase 2 clinical trials of osimertinib combined with bevacizumab vs. osimertinib alone as second-line targeted therapy for *EGFR* T790M-mutated NSCLC patients did not report a PFS benefit in the combination treatment arm [33, 34]. As a retrospective study of first-generation TKI combination regimens, the results also might have practical significance for guiding the administration of third generation TKI combination regimens.

There were a number of limitations to the present study. First, beside the small cohort of patients enrolled, its retrospective nature will likely have introduced selection bias. Therefore, the findings should be treated with a degree of caution until investigations with a larger cohort of patients, especially prospective studies, have been conducted. Second, the patients included in this study were from Henan Tumor Hospital, so the findings might not be applicable to the broader population of patients with BMs. Third, for the status of *EGFR* T790M after first-line treatment failure only 2 patients had a T790M mutation at first-line treatment with first-generation TKI, while the total amount of BMs, other comorbidities, and subsequent treatment regimens (e.g., oxitinib or immunotherapy) were not well documented, leading to a bias in OS estimation. Last but not least, the dose of bevacizumab (7.5 mg/kg) in our study was lower compared with prior clinical trials. However, the AVAiL study revealed there was no difference in OS times between bevacizumab 7.5 mg/kg and 15 mg/kg [34]. A dose of 7.5 mg/kg is used clinically under the Chinese medical insurance system.

## Conclusions

For *EGFR*m + NSCLC patients with BMs, an EGFR-TKI plus bevacizumab regimen significantly improved intracranial and ePFS times. Bevacizumab in combination with EGFR-TKI significantly prolonged the overall OS times without further improvement being provided by additional chemotherapy.

## Electronic supplementary material

Below is the link to the electronic supplementary material.

Supplementary Material 1

## Data Availability

The datasets used and/or analysed during the current study are available from the corresponding author.

## List of abbreviations

BMs: brain metastases
CNS: central nervous system
CR: complete response
CT: chemotherapy
EGFR: epidermal growth factor receptor
*EGFR*m+: *EGFR*-mutant positive
ePFS: extracranial PFS
iPFS: intracranial PFS
NSCLC: non-small cell lung cancer
ORRs: objective remission rates
OS: overall survival
PD: progressive disease
PFS: progression-free survival
PR: partial response
SD: stable disease
TKI: tyrosine kinase inhibitor
