# Supplementary material for: Clinical efficacy and prognosis analysis of treatment regimens for EGFR mutant non-small cell lung cancer and brain metastasis: a retrospective study
Source: BMC Cancer. 2023 Mar 30;23:289. doi: 10.1186/s12885-023-10744-2 (PMC10061743; doi:10.1186/s12885-023-10744-2)
Supplement: Supplementary file 1 — Supplementary Material 1 [file 12885_2023_10744_MOESM1_ESM.docx]

**Supplementary Table 1. Baseline characteristics of patients without antiangiogenic drugs group (group A+B) and with antiangiogenic drugs group (group C + D)**

| **Characteristic** | Group A + B | Group C + D | ***P* value** |
| --- | --- | --- | --- |
|  | (n = 139) | (n = 33) |  |
| **Age (years)** |  |  | 0.412 |
| < 70 | 114 (82.0%) | 25(75.8%) |  |
| ≥ 70 | 25 (18.0%) | 8 (24.2%) |  |
| **Gender** |  |  | 0.461 |
| Male | 56 (40.3%) | 11 (33.3%) |  |
| Female | 83 (59.7 %) | 22 (66.6%) |  |
| **Smoking status** |  |  | 0.291 |
| Yes | 33 (23.7%) | 4 (12.1%) |  |
| No | 116 (76.3%) | 29 (87.9%) |  |
| **ECOG PS** |  |  | 0.251 |
| 0-1 | 105 (75.5%) | 28 (84.8%) |  |
| 2 | 34 (24.5%) | 5（15.2%） |  |
| **EGFR mutation type** |  |  | 0.127 |
| Deletion in exon 19 | 70 (50.4%) | 21 (63.6%) |  |
| L858R | 63 (45.3%) | 9 (27.3%) |  |
| others | 6 (4.3%) | 3 (9.1%) |  |
| **Neurologic symptoms** |  |  | 0.396 |
| Yes | 44 (32.7%) | 13 (39.4%) |  |
| No | 95 (67.3%) | 20 (60.6%) |  |
| **Cranial radiation** |  |  | 0.436 |
| Yes | 18 (12.9%) | 6 (18.2%) |  |
| No | 121 (87.1%) | 27 (81.8%) |  |
| **Type of EGFR-TKIs** |  |  | 0.303 |
| Gefitinib | 97 (69.8%) | 26 (78.8%) |  |
| Icotinib | 42 (30.2%) | 7 (21.2%) |  |

Note: Group A + B: 1^st^ generation EGFR-TKIs monotherapy and 1^st^ generation EGFR-TKIs + pemetrexed plus cisplatin/carboplatin chemotherapy; Group C + D: 1^st^ generation EGFR-TKIs + bevacizumab and 1^st^ generation EGFR-TKIs + pemetrexed plus cisplatin/carboplatin chemotherapy + bevacizumab.

Abbreviation: TKI, tyrosine kinase inhibitor.

**Supplementary Table 2. Univariate and multivariate analysis of OS**

| **Independent risk factor** | | **Univariate analysis of OS** | | | **Multivariate analysis of OS** | | |
| --- | --- | --- | --- | --- | --- | --- | --- |
|  |  | ***P* value** | **HR value** | **95% CI** | ***P* value** | **HR value** | **95% CI** |
| **First line regimens** | Group C + D: Group A + B | 0.080 | 0.472 | 0.204-1.093 | 0.132 | 0.519 | 0.222-1.215 |
|  | Group B: Group A | 0.598 | 1.152 | 0.681-1.950 |  |  |  |
| **Age** | < 70: ≥ 70 | 0.006 | 0.474 | 0.279-0.806 | 0.039 | 0.555 | 0.317-0.972 |
| **Gender** | Female: male | 0.192 | 0.726 | 0.449-1.175 |  |  |  |
| **Intracranial symptoms** | No: Yes | 0.248 | 1.374 | 0.801-2.356 |  |  |  |
| **Smoking** | No: Yes | 0.020 | 0.532 | 0.313-0.907 | 0.255 | 0.717 | 0.4053-1.271 |
| ***EGFR* mutation** | Sensitive mutation: others | 0.006 | 0.333 | 0.152-0.729 | 0.072 | 0.451 | 0.190-1.073 |
|  | 21L858R: 19DEL | 0.484 | 1.195 | 0.725-1.969 |  |  |  |
| **Radiotherapy** | No: Yes | 0.058 | 2.250 | 0.972-5.210 | 0.076 | 2.158 | 0.923-5.049 |

Note: Group A + B: 1^st^ generation EGFR-TKIs monotherapy and 1^st^ generation EGFR-TKIs + pemetrexed plus cisplatin/carboplatin chemotherapy; Group C + D: 1^st^ generation EGFR-TKIs + bevacizumab and 1^st^ generation EGFR-TKIs + pemetrexed plus cisplatin/carboplatin chemotherapy + bevacizumab.

Abbreviation: OS, overall survival.

**Supplementary Table 3. Treatment related adverse event**

| **Types of adverse events** | **Grade** | **Group A**  **(n = 84)** | **Group B**  **(n = 55)** | ***P* value** | **Group C**  **(n = 15)** | **Group D**  **(n = 18)** | ***P* value** |
| --- | --- | --- | --- | --- | --- | --- | --- |
| **Paronychia** | Grade 1-2 | 9 (10.7%) | 7 (12.7%) | 0.716 | 1 (6.7%) | 1 (5.6%) | 1.000 |
|  | Grade 3-4 | 0 | 0 |  | 0 | 0 |  |
| **Erythema** | Grade 1-2 | 10 (11.9%) | 7 (12.7%) | 0.794 | 2 (13.3%) | 3 (16.7%) | 0.963 |
|  | Grade 3-4 | 2 (2.4%) | 0 |  | 0 | 0 |  |
| **Gastrointestinal reaction** | Grade 1-2 | 16 (19.0%) | 27 (49.1%) | **0.001** | 3 (20.0%) | 10 (55.6%) | **0.033** |
|  | Grade 3-4 | 3 (3.6%) | 3 (5.5%) |  | 0 | 1 (5.6%) |  |
| **Liver damage** | Grade 1-2 | 9 (10.7%) | 7 (12.7%) | 0.919 | 1 (6.7%) | 4 (22.2%) | 0.346 |
|  | Grade 3-4 | 1 (1.2%) | 0 |  | 0 | 0 |  |
| **Myelosuppression** | Grade 1-2 | 0 | 25 (45.5%) | **0.001** | 0 | 9 (44.4%) | **0.001** |
|  | Grade 3-4 | 0 | 2 (3.6%) |  | 0 | 1 (5.6%) |  |
| **Hypertension** | Grade 1-2 | 0 | 0 |  | 2 (13.3%) | 3 (16.7%) | 0.963 |
|  | Grade 3-4 | 0 | 0 |  | 0 | 0 |  |
| **Proteinura** | Grade 1-2 | 0 | 0 |  | 1 (6.7%) | 1 (5.6%) | 0.579 |
|  | Grade 3-4 | 0 | 0 |  | 1 (6.7%) | 0 |  |

Note: Group A: 1^st^ generation EGFR-TKIs monotherapy; Group B: 1^st^ generation EGFR-TKIs + pemetrexed plus cisplatin/carboplatin chemotherapy; Group C: 1^st^ generation EGFR-TKIs + bevacizumab; Group D: 1^st^ generation EGFR-TKIs + pemetrexed plus cisplatin/carboplatin chemotherapy + bevacizumab.

Abbreviation: TKI, tyrosine kinase inhibitor.
